# A novel digital approach to describe real world outcomes among patients with constipation

**DOI:** 10.1038/s41746-021-00391-x

**Published:** 2021-02-16

**Authors:** Allison Shapiro, Benjamin Bradshaw, Sabine Landes, Petra Kammann, Beatrice Bois De Fer, Wei-Nchih Lee, Robert Lange

**Affiliations:** 1 Evidation Health, Santa Barbara, CA USA; 2 Sanofi-Aventis, Consumer Healthcare, Frankfurt am Main, Germany; 3 Sanofi-Aventis Groupe, Global CHC Scientific & Clinical Platforms, Gentilly, France

**Keywords:** Digestive signs and symptoms, Outcomes research

## Abstract

Understanding day-to-day variations in symptoms and medication management can be important in describing patient centered outcomes for people with constipation. Patient Generated Health Data (PGHD) from digital devices is a potential solution, but its utility as a tool for describing experiences of people with frequent constipation is unknown. We conducted a virtual, 16-week prospective study of individuals with frequent constipation from an online wellness platform that connects mobile consumer digital devices including wearable monitors capable of passively collecting steps, sleep, and heart rate data. Participants wore a Fitbit monitoring device for the study duration and were administered daily and monthly surveys assessing constipation symptom severity and medication usage. A set of 38 predetermined day-level behavioral activity metrics were computed from minute-level data streams for steps, sleep and heart rate. Mixed effects regression models were used to compare activity metrics between constipation status (irregular or constipated vs. regular day), medication use (medication day vs. non-medication day) and the interaction of medication day with irregular or constipation days, as well as to model likelihood to treat with constipation medications based on daily self-reported symptom severity. Correction for multiple comparisons was performed with the Benjamini–Hochberg procedure for false discovery rate. This study analyzed 1540 enrolled participants with completed daily surveys (mean age 36.6 sd 10.0, 72.8% female, 88.8% Caucasian). Of those, 1293 completed all monthly surveys and 756 had sufficient Fitbit data density for analysis of activity metrics. At a daily-level, 22 of the 38 activity metrics were significantly associated with bowel movement or medication treatment patterns for constipation. Participants were measured to have fewer steps on irregular days compared to regular days (−200 steps, 95% CI [−280, −120]), longer periods of inactivity on constipated days (9.1 min, 95% CI [5.2, 12.9]), reduced total sleep time on irregular and constipated days (−2.4 min, 95% CI [−4.3, −0.4] and −4.0 min, 95% CI [−6.5, −1.4], respectively). Participants reported greater severity of symptoms for bloating, hard stool, difficulty passing, and painful bowel movements on irregular, constipation and medication days compared to regular days with no medication. Interaction analysis of medication days with irregular or constipation days observed small increases in severity compared to non-medication days. Participants were 4.3% (95% CI 3.2, 5.3) more likely to treat with medication on constipated days versus regular. No significant increase in likelihood was observed for irregular days. Daily likelihood to treat increased for each 1-point change in symptom severity of bloating (2.4%, 95% CI [2.0, 2.7]), inability to pass (2.2%, 95% CI [1.4, 3.0]) and incomplete bowel movements (1.3%, 95% CI [0.9, 1.7]). This is the first large scale virtual prospective study describing the association between passively collected PGHD and constipation symptoms and severity at a day-to-day granularity level. Constipation status, irregular or constipated, was associated with a number of activity metrics in steps and sleep, and likelihood to treat with medication increased with increasing severity for a number of constipation symptoms. Given the small magnitude of effect, further research is needed to understand the clinical relevance of these results. PGHD may be useful as a tool for describing real world patient centered experiences for people with constipation.

## Introduction

Constipation, a condition characterized by infrequent defecation, hard stools or straining, occurs commonly, particularly among women and the elderly^1,2^. While constipation can be transient, especially for people with recurrent or chronic symptoms, it negatively impacts their quality of life, and the downstream economic effects of lost workdays, decreased productivity, and increased health services use can be substantial^3,4^. A number of prescription and non-prescription constipation remedies exist, with non-prescription use playing an important role. In general, these medications have demonstrated efficacy in clinical trials^5,6^. Real world data (RWD), conventionally collected through health records, claims data or prescribing data, reflects individual or population usage in uncontrolled settings, and serves as an important tool for drug monitoring or discovery of new therapeutic uses^7,8^.

However, describing real-world treatment patterns of people with constipation is challenging. Over the counter medication use limits the utility of claims based or pharmacy-based data. Information on constipation management typically relies on cross sectional surveys, which are subject to recall and recency bias^9,10^. Validated activity surveys such as the International Physical Activity Questionnaire (IPAQ), do not capture other behavioral dimensions such as sleep activity^11,12^. Clinical trials for constipation agents do not provide insight into real-world treatment patterns or patient experiences, and pharmacy or prescribing data typically do not reflect management strategies in the outpatient setting^13^. For people with constipation, there is an opportunity for exploring new ways for generating real world evidence to better characterize patient experiences and thus understand the patient journey.

Digital Patient Generated Health Data (PGHD), a recently introduced technology, has become available with the widespread availability of digital mobile devices, and can potentially fill the information gaps left by conventional sources of real-world data^14,15^. Wearable devices in particular that track physical activities or physiologic parameters like heart rate allow for the passive inference of behavioral activity patterns with time granularities not possible with conventional RWD^16–18^. Though still in the early stages of widespread clinical use, PGHD could potentially be used to gain more nuanced data on patient centered outcomes, and serve as passive health diaries with information to guide medication approaches personalized to the individual user^19^.

We report the first virtual large-scale prospective study to evaluate the utility of digitally collected active and passive PGHD for describing and linking symptom characteristics and medication use patterns for people with frequent constipation. For our primary objective, we hypothesized that differences in behavior as recorded from wearable activity trackers may be used to discern differences in these parameters between days with reported constipation, and days where individuals report regular bowel movement patterns. As a secondary objective, we also explored digital daily diaries for describing symptom severity and its relationship to medication use and treatment preferences for constipation episodes.

## Results

### Study population descriptive characteristics

**Fig. 1:**
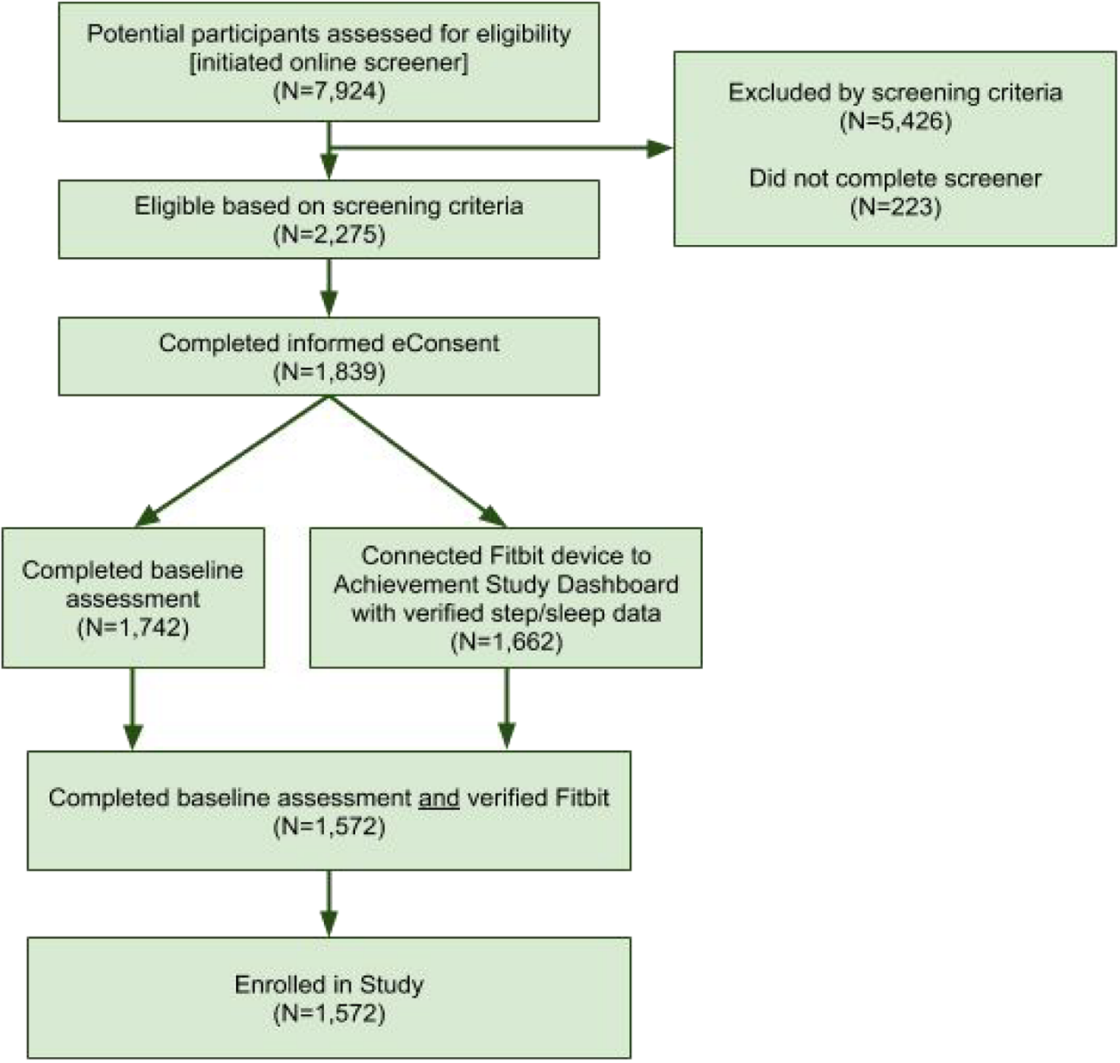
Flow diagram of participants from recruitment to study enrollment. Flow of participants (starting at top) from initial eligibility screening to informed consent to to enrollment completion is shown. Participants were considered enrolled if they completed the baseline assessment and connected a Fitbit device after consenting to the study.

Figure 1 shows the flow of participants from recruitment to enrollment. A total of 7924 individuals were screened for study eligibility, of whom 5426 individuals (68.5%, 5426/7924) did not meet screening criteria, 223 individuals (2.8%, 223/7924) did not complete the screening questionnaire, which resulted in 2275 potential participants (28.7%, 2275/7924) who were eligible based on screening criteria. Among those eligible, 1839 individuals (80.8%, 1839/2275) completed eConsent procedures. After completing informed consent, 1572 participants (85.5%, 1572/1839) completed the final steps required for enrollment, completion of baseline assessment, and connection of a Fitbit device, and were then enrolled into the study. Of those, 32 participants did not complete any of the daily surveys, and were removed, leaving 1540 enrolled persons in the analysis. Analysis of management approaches for constipation was conducted on those individuals who completed all the monthly surveys (*n* = 1293 or 82% of enrolled), while the behavioral activity analysis was performed on those individuals who satisfied the previously described data density criteria (*n* = 756 or 48% of enrolled).

Study participants were relatively young (mean age 36.6), and mostly female (72.8%), Caucasian (88.8%) and well educated (69.6% with at least a college degree). In terms of general management approaches, most participants (34%) reported a combination of lifestyle changes with waiting to see if symptoms improved. Only a small proportion of participants regularly used constipation medications (7.7%) (Table 1). Fitbit data coverage was generally high for the enrolled participants (mean coverage 82.2% of 112 study days for steps), and even higher for the Behavioral Populations subcohort. Not all participants had Fitbit devices capable of heart rate monitoring, which explains the lower coverage rates for heart rate features (67.4% in the enrolled population).

**Table 1:** Demographic and descriptive characteristics of the population.

Characteristic	Daily population	Behavioral population	Monthly population
Number	1540	756	1293
Mean age in years (SD)	36.6 (10.0)	37.5 (10.0)	36.8 (10.0)
Mean BMI in kg/m^2^ (SD)	29.7 (6.8)	29.9 (6.8)	29.6 (6.7)
Female gender, *n* (%)	1121 (72.8)	516 (68.3)	959 (74.2)
*Education, n (%)*^a^			
≥College degree	1070 (69.5)	540 (71.4)	914 (70.7)
Some college	308 (20.0)	142 (18.8)	253 (19.6)
Trade or vocational	68 (4.4)	28 (3.7)	57 (4.4)
High school graduate	88 (5.7)	42 (5.6)	66 (5.1)
No high school	5 (0.3)	3 (0.4)	2 (0.2)
*Race, n (%)*			
White	1367 (88.8)	683 (90.3)	1155 (89.3)
African American	105 (6.8)	49 (6.5)	83 (6.4)
Asian	71 (4.6)	25 (3.3)	58 (4.5)
American Indian or Alaska Native	29 (1.9)	11 (1.5)	23 (1.8)
Native Hawaiian or Pacific Islander	10 (0.6)	5 (0.7)	7 (0.5)
Other	36 (2.3)	14 (1.9)	28 (2.2)
*Mean Pct of study days with data (%)*			
Survey	67.2%	70.8%	76.1%
Steps	82.2%	96.0%	84.2%
Sleep	75.9%	88.7%	78.5%
Heart rate	67.4%	74.0%	68.7%
*Management approaches, n (%)*			
Lifestyle changers	–	–	97 (8%)
Immediate treaters	–	–	207 (16%)
Lifestyle + Waiting	–	–	438 (34%)
Waiters	–	–	279 (22%)
Other	–	–	272 (21%)
*Medication approaches, n (%)*			
As needed	–	–	367 (28.4)
Regular	–	–	99 (7.7)
Non-user	–	–	403 (31.2)
Other	–	–	424 (32.8)

Daily population refers to individuals who completed the daily surveys. Behavioral population refers to a subset of the daily population who satisfied data density requirements for the analysis of wearable activity metrics. Monthly population refers to individuals who completed at least one monthly survey for the analysis of medication and management approaches.

^a^Education categories are missing one participant who did not complete this question in the baseline questionnaire.

### Relation of daily behavioral activity to constipation symptoms and medication use

**Fig. 2:**
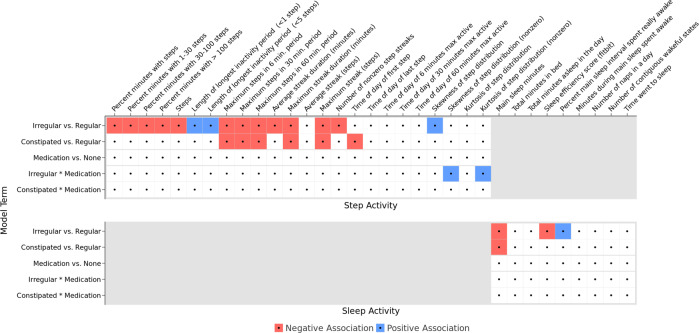
Regression model results for steps and activity features. Overview of the regression model results for the five different comparisons of bowel movement or medication use patterns to steps and sleep activity features. Significant results are shown in color, with red denoting a negative association and blue denoting a positive association. Among the models for heart rate features (not shown) significant associations were only observed for 95th percentile heart rate, which was negatively associated with both irregular and constipated days.

22 of the 38 daily activity features were found to be significantly associated with different patterns of bowel movements. Most of these associations centered around constipation status, where participants tended to be less active (in daily steps or sleep activity) on irregular or constipated days versus regular days. Only two steps features (skewness and kurtosis of step distribution) were found to be associated with the interaction of irregular days with medication use, otherwise medication days were not associated with activity features. Active heart rate, computed as the 95th percentile of heart rate for the day, was associated with irregular and constipated BM days when compared to regular BM days. The remaining heart rate features were not significant after accounting for multiple comparisons (Fig. 2).

Compared with regular days, days with irregular BM patterns saw fewer steps (−200 steps per day, 95% CI [−280, −120]) and longer periods of inactivity (9.1 min additional minutes, 95% CI [5.2, 12.9]). There was a small reduction in sleep efficiency, defined as the proportion of time asleep to time in bed, on irregular days compared to regular BM days (−0.1% 95% CI [−.19%, −.01%]). Participants also had a small reduction in total minutes asleep on irregular and constipation BM days compared to regular BM days (−2.4 min, 95% CI [−4.3, −0.4] and −4 min, 95% CI [−6.5, −1.4], respectively). Active heart rate, a proxy of the time a person spends engaged in physical activity, was also lower on irregular and constipation BM days when compared to regular days—consistent with the findings of decreased steps activity (Table 2).

**Table 2:** Activity features and patterns of bowel movement or treatment approach.

	Intercept	Irregular – Regular	Constipated –Regular	Medication –No Medication	Irregular x Medication	Constipation x Medication
	Severity on regular days, no medication	Change on irregular days	Change on constipated days	Change on medication days	Change on irregular days with medication	Change on constipated days with medication
*Regression coefficient estimates [95% CI]*						
Total steps	8887 [8627, 9147]	−200* [−280, −120]	−96 [−200, 8]	−102 [−375, 171]	−56 [−396, 284]	−96 [−452, 260]
Maximum consecutive steps	1764 [1665, 1863]	−57* [−92, −22]	−94* [−139, −48]	95 [−24, 214]	−46 [−194, 103]	−64 [−219, 91]
Longest inactive period (minutes)	533 [525, 541]	9.1* [5.2, 12.9]	0.1 [−5.0, 5.084]	9.871 [−3.2, 23.0]	−7.423 [−23.9, 9.1]	11.239 [−5.9, 28.4]
Nightly sleep duration (minutes)	410 [404, 415]	−2.4* [−4.3, −0.4]	−4.0* [−6.5, −1.4]	−0.15 [−6.7, 6.4]	3 [−5.2, 11.3]	2.1 [−6.5, 10.8]
Sleep efficiency score (%)	91.12 [90.39, 91.85]	−0.10* [−0.19, −0.01]	0.033 [−0.08, 0.15]	0.023 [−0.27, 0.31]	0.08 [−0.29, 0.44]	−0.16 [−0.54, 0.22]
Active HR (95 percentile of daily HR; BPM)	103.9 [102.3, 103.7]	−0.27* [−0.48, −0.07]	−0.46* [−0.73, −0.19]	0.23 [−0.47, 0.93]	−0.83 [−1.68, 0.02]	−0.73 [−1.63, 0.18]

Magnitude of changes in selected activity features estimated from regression models for each comparison type of bowel movement or treatment pattern. Each row presents the results of a different model, the dependent variable of the model (an activity feature) is shown in the first cell of that corresponding row. Each column presents a different regression coefficient. The second column, Intercept, shows the model intercept or the baseline measurement, which is the activity observed at the reference level of the predictor variables (regular days, no medication). Columns 3-7 reflect changes with respect to the baseline measure.

^*^Statistically significant with *q*-value < 0.05.

As expected, participants reported significant changes in symptom severity on irregular and constipated BM days when compared to regular BM days. Medication days compared to non-medication days were also associated with significant increases in symptom severity. Medication days during irregular stool days were not associated with significant changes in bloating and painful BM severity (Table 3).

**Table 3:** Symptom severity and patterns of bowel movement or treatment approach.

	Intercept	Irregular–regular	Constipated–regular	Medication–no medication	Irregular × medication	Constipation × medication
	Severity on regular days, no medication	Change on irregular days	Change on constipated days	Change on medication days	Change on irregular days with medication	Change on constipated days with medication
*Regression coefficient estimates [95% CI]*						
Bloating severity	0.47 [0.45, 0.5]	0.38* [0.37, 0.39]	0.70* [0.69, 0.72]	0.30* [0.27, 0.33]	0.00004 [−0.04, 0.04]	0.14* [0.10, 0.18]
Hard stool severity	0.21 [0.19, 0.22]	0.35* [0.34, 0.36]	1.5* [1.48, 1.52]	0.11* [0.08, 0.15]	0.11* [0.06, 0.15]	0.06* [0.01, 0.12]
Painful BM severity	0.21 [0.19, 0.23]	0.38* [0.37, 0.39]	0.79* [0.78, 0.81]	0.17* [0.13, 0.20]	0.03 [−0.008, 0.07]	0.14* [0.09, 0.18]
Difficulty passing severity	0.41 [0.39, 0.44]	0.47* [0.45, 0.48]	1.36* [1.35, 1.38]	0.18* [0.14, 0.21]	0.10* [0.06, 0.15]	0.14* [0.08, 0.19]

Magnitude of changes in self-reported daily symptom severity estimated from regression models for each comparison type of bowel movement or treatment pattern. Each row presents the results of a different model, the dependent variable of the model (a symptom) is shown in the first cell of that row. Each column presents a different regression coefficient. The second column, Intercept, shows the model intercept or the baseline measurement, which is the activity observed at the reference level of the predictor variables (regular days, no medication). Columns 3–7 reflect changes with respect to the baseline measure. Symptom severity was rated on a 0–4 scale.

^*^Indicates statistically significant result (*q*-values < 0.05).

**Fig. 3:**
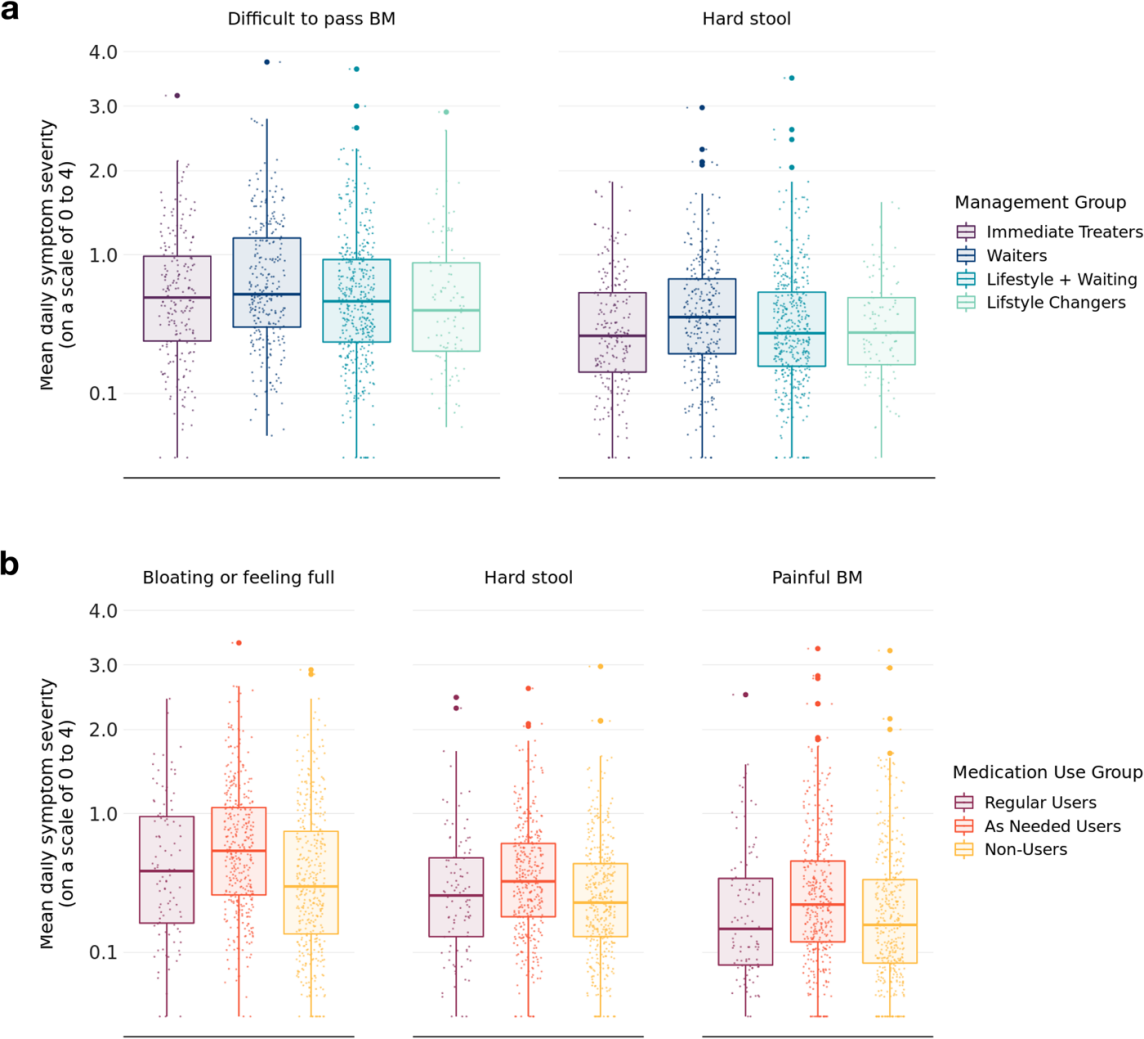
Boxplots of symptom severity. **a** A boxplot of symptom severity on self-reported management approach from the monthly surveys. **b** A boxplot of symptom severity on medication use approach from the monthly surveys. Symptoms scores in each group are on a 0–4 scale with 4 as the most severe symptom. Each boxplot shows the median (center line) and inter-quartile range (box bounds) and the largest (top whisker) and smallest (bottom whisker) values within 1.5* interquartile. Only significant results are shown.

From the monthly surveys, we observed more severe symptoms among people who tended to wait to manage their symptoms. Waiters reported greater severity of difficulty passing and hard stools when compared to Lifestyle changers and Immediate treaters, while people who used medications as needed had greater severity of bloating, hard stool, and painful bowel movements (Fig. 3).

Upper edge represents the 75th percentile, while the lower edge shows the 25th percentile. Only significant results are shown. Symptom severity is based on a 4-point scale. In a) Waiters differed significantly from Lifestyle changers in terms of symptom severity for difficulty passing and hard stool. In b) As needed users differed significantly from non-users in terms of bloating, hard stool and painful BM.

The coefficients in Table 4 show the increase in likelihood of treating for each 1-point increase in symptom severity score. The largest increase in treatment likelihood was due to participants reporting constipated BM (4.3% increase compared to regular days, 95% CI [3.2, 5.3]), and on days with increasing severity of bloating, difficulty passing stool, or pain. For all symptom and bowel movement parameters, the estimated coefficients were directionally larger for females as compared to males. This indicates that generally increased symptoms, as well as self-reported bowel patterns of constipation directionally increase the probability of treatment more for females as compared to males. The magnitude of this difference appeared to be especially large for bloating (2.6% likelihood to treat vs 1.5% for women vs. men) as well as self-reported constipation bowel movement pattern (4.5% likelihood to treat vs. 3.2% likelihood to treat for women vs. men).

**Table 4:** Estimated percent increase in likelihood of using a constipation medication.

	% Likelihood increase** [95% CI]		
Parameter	Full cohort	Female only	Male only
Bowel pattern: none of the above	−3.3 [−6.7, 0.1]	–	–
Bowel pattern: irregular	-0.2 [−1.0, 0.5]	−0.2 [−1.1, 0.6]	0.3 [−0.8, 1.4]
Gas	0.1 [−0.3, 0.5]	0.2 [−0.3, 0.7]	−0.2 [−0.8, 0.4]
Nausea	0.2 [−0.5, 0.9]	0.1 [−0.7, 0.9]	0.3 [−1, 1.5]
Hard stool	0.3 [−0.2, 0.7]	0.2 [−0.4, 0.8]	0.4 [−0.3, 1.2]
Painful BM	0.4 [−0.1, 0.9]	0.4 [−0.2, 1]	0.4 [−0.6, 1.5]
Difficulty passing	0.6* [0.1, 1.0]	0.6* [0, 1.2]	0.6 [−0.1, 1.2]
Cramps	0.6* [0, 1.3]	0.6 [−0.2, 1.3]	0.6 [−0.5, 1.7]
Pain	0.9* [0.2, 1.6]	1* [0.2, 1.8]	0.1 [−1.2, 1.4]
Incomplete BM	1.3* [0.9, 1.7]	1.4* [0.9, 1.8]	1* [0.2, 1.8]
Unable to pass	2.2* [1.4, 3.0]	2.1* [1.2, 3]	2.3* [0.9, 3.7]
Bloating	2.4* [2.0, 2.7]	2.6* [2.2, 3.1]	1.5* [0.8, 2.1]
Bowel pattern: constipated	4.3* [3.2, 5.3]	4.5* [3.3, 5.7]	3.2* [1.1, 5.3]

The percent increase in likelihood of using a constipation medication for each unit change in daily symptom severity is shown for the full daily survey cohort, and then based on self-reported gender. 95% confidence limits are shown in the brackets.

^*^Indicates statistically significant result (*q*-values < 0.05).

^**^Increases are for a change in 1-point of symptom severity.

## Discussion

Despite some non-interventional studies suggesting that constipation and irregularity were associated with less active lifestyles^20,21^, empirical investigations into this claim do not always produce consistent evidence. For example, whereas one survey-based study reported no relationship between physical activity and self-reported constipation among 1069 respondents^22^, two other large-scale survey studies of similar design but comprising nearly 40,000 and over 60,000 women found lower prevalence of constipation among women who reported some degree of physical activity versus a sedentary lifestyle^23,24^.

Although there have been previous studies of activity and constipation, this is the first study that utilized commercially available activity trackers to quantify the association between self-reported bowel patterns, laxative utilization, and patterns of behavior related to activity and sleep at a daily level^25,26^. The 4-month longitudinal nature of this study, in combination with its scale of more than 1500 participants with approximately 750 wearing activity tracking devices recording minute level step, sleep, and heart rate measures, allowed for the first time the opportunity to examine how constipation symptoms as well as constipation treatment patterns were associated with daily behavior.

This body of research revealed several associations of note. The baseline steps activity in our cohort is similar to baseline steps in other settings^27^. Days with self-reported constipation and irregularity were negatively associated with many variables that quantified the level and distribution of daily activity. Although the magnitudes of the behavioral changes with regards to activity observed in this study are small, their trend is consistent with other research that shows that more severe disease states are associated with fewer steps per day^28,29^. The smaller effect of our findings may be explained by the focus on constipation episodes that are of shorter duration. Additionally, the study participants, who reflect more real world experiences, may have more heterogeneity in constipation symptoms than those who participate in clinical trials. Constipation and irregularity showed significant association in 22 of 38 models with decreased sleep and step activity. Specifically, activity features capturing sleep quality and duration, as well as activity features capturing the quantity and timing of daily steps showed association on days with constipation and irregularity. Given the small magnitude of the effects observed in this study, further research is needed to understand the clinical relevance of the results. More stringent inclusion and exclusion criteria might lead to more pronounced effects in future studies using RWD.

It was also found that individuals reported increased symptom severity on days with constipation or irregularity and use of laxative medications compared to days with constipation status being constipated or irregular but abstinence from medication. This finding may be interpreted as evidence that on average individuals opt for treatment once their symptoms surpass an internal symptom threshold, rather than treating as soon as any symptoms manifest. The results of the daily self-reported behaviors are supported by participants’ perceptions of their own behaviors from the monthly surveys—“Waiters” and people who took medications “As-needed” noted more symptom severity, and likelihood to take medications increased with increasing symptom severity.

In line with previous reports, positive associations between self-reported daily constipation and individual’s likelihood to treat were observed. Furthermore, a positive association of laxative treatment and bloating has been seen. Bloating has been known to be a symptom of constipation but has not been widely associated with an increased likelihood for laxative treatment. This observation may suggest that individuals deem laxative medications more appropriate for bloating relief than previously known. Further research is needed to better understand the factors that motivate patients to use laxative medications.

The study has limitations. The study cohort consists predominantly of well educated, comparably young Caucasian women with access to mobile wearable devices, which limits generalizability to other populations. To reduce the participant burden, we used shorter daily surveys with questions customized to assess constipation severity and medication use instead of known validated surveys, which may have reduced the observable effect size. One early study has suggested that constipation in elderly people is associated with lack of activity, which may also be important when looking at the small magnitude of effects observed in this trial^29^. Another limitation is that we did not specifically exclude participants for using older versions of the Fitbit device. However, the data requirement to have minute-level granular data for steps and sleep would have removed from analysis people with older devices that did not have this capability. The requirement for high density data from the Fitbit devices may have selected participants who are more engaged in wellness behaviors. Fitbit devices may also be inaccurate when compared to conventional gold standards for steps and sleep, though the devices themselves have been shown to be internally reliable and consistent^30–32^. Fitbit devices may measure steps or sleep differently depending on the location of use^33^, but this fixed effect of location is accounted for in the mixed-effects models used for the studies. Finally, the use of only three categories to describe BM days makes it difficult to translate the results to prior studies that use standard diagnostic criteria like the Rome criteria^34^.

In summary, this study is the largest virtual study of activity and constipation using passively and actively collected PGHD with more than 100 million digital activity data points. The study allowed us to measure activity based associations with constipation and medication use at minute-level granularity over extended periods of time, which is not possible with conventional real-world approaches^35^. The study findings were also consistent with prior research on the effects of physical activity on symptoms of constipation.

## Methods

### Study design and Platform

A 16-week prospective and completely virtual study was conducted with a novel study platform (Achievement Studies, Evidation Health Inc., San Mateo, CA). The Achievement study platform is available as a mobile application for both Android and iOS operating systems, with functionality to connect consumer mobile devices and third-party digital diary apps, as well as enable users to participate and engage in clinical research efforts^36,37^. The Achievement community, which numbers more than 3 million, consists of adults (age ≥ 18) residing in the United States with members in all 50 states.

### Study population

Study participants were recruited from the Achievement platform with an email or within-app offer to participate in the study, targeting adults (age ≥ 18) residing in the United States. Additional inclusion criteria for participation were:

- Self-reported constipation, irregular bowel movements, and/or hard dry stool at least once every 2 months.
- Self-reported use of at least one over-the-counter constipation medication in the past 3 months.
- Access to a Fitbit activity tracker capable of collecting steps, heart rate or sleep data that was connected to the Achievement platform and willing to wear the tracker daily during the day and at night for the entire study.
- Willingness to respond to daily short questionnaires and report whether they experienced constipation symptoms the previous day.

Potential participants were excluded from participation if they self-reported a diagnosis of Inflammatory Bowel Disease (Ulcerative Colitis and Crohn’s Disease), irritable bowel syndrome, gastrointestinal malignancies, or chronic constipation. Potential enrollees into the study were determined by sending a screening questionnaire to interested participants. Individuals who satisfied inclusion and exclusion criteria were then confirmed to have functioning Fitbit devices that were connected to the Achievement platform. All enrolled participants completed an online informed consent form agreeing to study protocols. The study was approved by Solutions Institutional Review Board (Yarnell, AZ, USA).

### Survey data variables

Participants enrolled in the study completed three survey questionnaire types, each with different time frequencies. As described above, a single baseline survey was administered to potential participants to determine eligibility for the study, and to gather baseline demographic data as well as information about medication use and constipation symptoms. A monthly survey (every 4 weeks) was administered to enrolled participants (four surveys total per participant) asking them to describe self-reported management patterns for constipation and quality of life metrics. Lastly, a daily survey asking about constipation symptoms and medication use, started within 24 h of enrollment, was administered to every participant for the duration of the study (16 weeks or 112 days total).

Demographic data captured in the baseline survey include date-of-birth, gender, race and ethnicity, highest educational level, and comorbid conditions. Body mass index (weight in kg per meter^2^) is computed from self-reported height and weight data. Age was determined by the date of birth as reported at the time of enrollment.

In the monthly survey, participants were asked about management approaches on two dimensions in the prior month for symptoms of constipation/irregularity. The first dimension focused on general management approaches, which were categorized as Treat with medication immediately, Treat with lifestyle/dietary changes, Watchful waiting, or No treatment done. The second addressed the frequency of medication use, with options for Regular use of medications, As-needed use, and no-use of medications. For both of these dimensions, the 4 monthly responses were aggregated over the entire study period for each participant to assign him/her to treatment categories. General management groups were: Waiters (all four responses Watchful waiting), Lifestyle Changers (all four responses lifestyle/dietary changes), Lifestyle + Waiting (any combination of Watchful waiting and lifestyle/dietary changes), or Immediate Treaters (any response of medication immediately). Medication frequency groups were: Regular user (all four responses regular), As needed user (minimum one response As-needed) or Non-user (all four responses no-use). People who did not fit into one of the aggregated categories were labeled as “Other”.

For the daily surveys, participants were asked about bowel habits for the previous day, which were labeled into mutually exclusive categories of normal, irregular, constipated, or none of the above. All participants were also asked to rate on a 4-point severity scale symptom dimensions of bloating, hard stool, painful bowel movement, or difficulty passing stool. Those who reported Irregular or Constipated days were asked to rate five additional symptoms of incomplete bowel movements, periumbilical cramps, sub-umbilical cramps, flatulence and nausea. Finally, participants were also asked about medication use to manage constipation symptoms for the previous day. A list of common over-the-counter (OTC) medications, including stool-softeners, laxatives, suppositories, stool bulk, and fiber supplements was provided for the participant to label with binary Yes or No responses if the medication(s) were used. Daily survey questions on constipation severity and medication use were drawn from a bank of questions that had been used by Sanofi-Aventis for consumer-focused research. Participant days for which any medication was used was labeled a medication day (binary Yes or No) for that participant.

### Patient generated activity tracker data

The Achievement platform uses the Fitbit application programming interface (API) to pull data on steps, heart rate and sleep for each participant at minute level granularity (e.g. steps per minute, heart beats per minute, sleep states of asleep, restless or awake). The Fitbit API includes for each sleep state a binary (True/False) designation for the “main” sleep period of the day.

Each activity data stream (steps, heart rate, and sleep) was then aggregated into a set of daily activity features engineered in collaboration with a medical domain expert. The features were designed to capture behavioral episodes that reflected a priori beliefs about the effect that constipation might have on these different types of activity. Table 5 shows a summary of the activity features along with a brief description. A total of 38 daily activity features were engineered (24 steps, 9 sleep, and 5 heart rate features). Skewness and kurtosis features for steps and heart rate were computed for each daily distribution. Features over a specific duration (e.g., 5, 6, 30, or 60 min) were determined with a sliding window of that duration over the entire day. The Sleep Efficiency score is provided from the Fitbit API and is determined by the proportion of time asleep (main sleep at night) to the time spent in bed.

**Table 5:** Fitbit derived behavioral activity features.

Steps activity features			Sleep activity features	Heart rate features
Percent minutes with steps	Maximum steps in 30-min period	Time of day of last step	Main sleep minutes	Resting heart rate (beats per minute)
Percent minutes with 1–30 steps	Maximum steps in 60-minute period	Time of day of 6 min max active	Total minutes spent in bed	Skewness of heart rate
Percent minutes with 30–100 steps	Average streak duration (minutes)	Time of day of 30 min max active	Total minutes asleep in day	Kurtosis of heart rate
Percent minutes with >100 steps	Maximum streak duration (minutes)	Time of day of 60 min max active	Sleep efficiency score (Fitbit)	Max rolling 5-min average
Steps taken per day	Average streak (steps)	Skewness of step distribution (all values)	Percent main sleep interval spent really awake	95th percentile of heart rate
Length of longest inactivity period (<1 step)	Maximum streak (steps)	Skewness of nonzero step distribution	Minutes during main sleep spent awake	
Length of longest inactivity period (<5 step)	Number of nonzero step streaks	Kurtosis of step distribution (all values)	Number of naps in a day	
Maximum steps in 6-min period	Time of day of first step	Kurtosis of nonzero step distribution	Number of contiguous wakeful states	
			Time went to sleep	

Data streams from the Fitbit API were downloaded at minute-level granularity, and then aggregated into day-level features with input from a medical domain expert.

Analysis using PGHD (activity data and daily survey responses) was conducted on a subgroup of the study cohort with high density activity tracker data (the Behavioral Analysis Population). To define this subgroup, we followed a three-step process that has been previously used:^38,39^

1. Fitbit wear time for each participant computed based on available minute-level data,
2. Any day with wear time <10 h was labeled as an invalid day, and
3. Removal of all individuals with ≥7 consecutive invalid days.

### Statistical methods

Linear mixed-effects regression was used to estimate the amount of variance in daily activities or constipation severity that could be explained by bowel movement (BM) patterns and medication use. The regression models took the form:

1 $$\begin{array}{l}Y_{{\rm{i,t}}}\sim \beta _1{\rm{medication}}_{{\rm{i,t}}} + \Sigma _{\rm{j}}\beta _{\rm{j}}BM{\rm{pattern}}_{{\rm{j,i,t}}}\\\,\,\,\,\,\,\,\,\,\,\,\,\, + \Sigma _{\rm{k}}\beta _{\rm{k}}{{\rm{medication}}_{{\rm{i,t}}}}^\ast BM{\rm{pattern}}_{{\rm{j,i,t}}} + u_{\rm{i}},\end{array}$$

where,

1. *Y*_i,t_ is a day level activity feature or day level self-report of constipation severity for individual *i* (for *i* = 1, 2, 3, …, *n*; where *n* is the number of participants in the Behavioral Analysis Population), on day *t* (for *t* = 1, 2, 3, …, 112th day of study)
2. medication_i,t_ is a binary variable that is 1 for individual *i* on day *t* if a laxative is reported taken, else 0
3. *BM*pattern_j,i,t_ is a dummy-coded variable indicating if individual *i* reported being constipated or irregular as opposed to regular on day *t,* with regular BM pattern being the reference state.
4. *u*_i_ is a random intercept which is allowed to vary for each participant.

For each dependent variable, significance of association was determined on the β-coefficient for five comparisons—irregular vs. regular days, constipated vs. regular days, medication vs. no-medication days, and the interaction terms for irregular × medication days and constipation × medication days. Daily observations in the model were excluded for both the activity feature and symptom severity models if the participant reported their BM pattern to be “None of the above”, failed to respond to the symptom severity, or if there was no response to the medication use question.

In an effort to better understand what drives individuals to utilize laxative medications, an individual’s likelihood of treating on a particular day (used laxatives) given self-reported daily bowel movement pattern as well as reported daily symptom severity was modeled. Using a fixed-effects panel regression, the following linear probability model was estimated:

2 $${\rm{Medication}}_{{\rm{i,t}}} = \beta _0 + \Sigma _{\rm{j}}\beta _{\rm{j}}{\rm{symptom}}_{{\rm{j,i,t}}} + \Sigma _{\rm{k}}\beta _{\rm{k}}BM{\rm{pattern}}_{{\rm{k,i,t}}},$$

where:

1. medication_i,t_ is a binary variable that is 1 for individual *i* on day *t* if a laxative is reported taken, else 0,
2. symptom_j,i,t_ is an ordinal symptom severity indicator for individual *i* on day *t* with levels ranging from 0 to 4 for each of the *j* symptom types (e.g., gas, difficulty passing, etc.) with 0 indicating no symptoms, and 4 indicating most severe symptoms,
3. *BM*pattern_k,i,t_ is a dummy variable indicating if individual *i* reported being constipated, irregular, or “None of the above” as opposed to regular on day *t,* with regular BM pattern being the reference state.

We looked at daily responses to symptom severity across treatment approaches categories (general management and medication frequency) as determined from the monthly surveys. Comparison of means across different groups was conducted with Kruskal–Wallis (non-parametric) ANOVA tests or the Wilcoxon rank sum test when appropriate. For all the analyses, we adjusted *p*-values (*q*-value ≤0.05 significant) for multiple comparisons with the Benjamini–Hochberg procedure for false discovery rate (FDR). All statistical tests were two-sided.

### Reporting summary

Further information on experimental design is available in the Nature Research Reporting Summary linked to this paper.

## Supplementary information

Reporting Summary

## Data Availability

Qualified researchers may request access to the data and related study documents including the study report, study protocol with any amendments, blank case report form, statistical analysis plan, and dataset specifications. Further details on Sanofi’s data sharing criteria, eligible studies, and process for requesting access can be found at https://www.clinicalstudydatarequest.com.

## Code availability

The code that supports the findings of this study are available from the corresponding author upon reasonable request. Analysis to process and analyze the data was generated with Python 3 and the R programming language.
