# Author Correction: A novel digital approach to describe real world outcomes among patients with constipation

**DOI:** 10.1038/s41746-021-00434-3

**Published:** 2021-03-24

**Authors:** Allison Shapiro, Benjamin Bradshaw, Sabine Landes, Petra Kammann, Beatrice Bois De Fer, Wei-Nchih Lee, Robert Lange

**Affiliations:** 1 Evidation Health, Santa Barbara, CA USA; 2 Sanofi-Aventis, Consumer Healthcare, Frankfurt am Main, Germany; 3 Sanofi-Aventis Groupe, Global CHC Scientific & Clinical Platforms, Gentilly, France

**Keywords:** Digestive signs and symptoms, Outcomes research

Correction to: *npj Digital Medicine* 10.1038/s41746-021-00391-x, published online 16 February 2021

The original version of the published Article mistakenly omitted the variable names in the description of the statistical methods. To improve clarity, the variable names have been added to the description of the statistical methods as follows:

1 $$Y_{\mathrm{i,t}}\sim \beta _{1}{\mathrm{medication}}_{{\mathrm{i,t}}} + \mathop{\Sigma}\nolimits _{\mathrm{j}}\beta _{\mathrm{j}}{BM}{\mathrm{pattern}}_{{\mathrm{j,i,t}}}+ \mathop{\Sigma}\nolimits _{\mathrm{k}}\beta_{\mathrm{k}}{\mathrm{medication}}_{{\mathrm{i,t}}}\; {{\,}^{\ast}}\; {{BM}}{\mathrm{pattern}}_{{\mathrm{j,i}},{\mathrm{t}}} + u_{\mathrm{i}}$$

Where:

1. *Y*_i,t_ is a day level activity feature or day level self-report of constipation severity for individual *i* (for *i* = 1, 2, 3, …, *n*, where *n* is the number of participants in the Behavioral Analysis Population), on day *t* (for *t* = 1, 2, 3, …, 112th day of study)
2. medication_i,t_ is a binary variable that is 1 for individual *i* on day *t* if a laxative is reported taken, else 0
3. *BM*pattern_j,i,t_ is a dummy-coded variable indicating if individual *i* reported being constipated or irregular as opposed to regular on day *t*, with regular BM pattern being the reference state
4. *u*_i_ is a random intercept that is allowed to vary for each participant

2 $${\mathrm{Medication}}_{\mathrm{i,t}} = \beta _0 + \mathop{\Sigma}\nolimits _{\mathrm{j}}\beta _{\mathrm{j}}{\mathrm{symptom}}_{\mathrm{j,i,t}} + \mathop{\Sigma}\nolimits _{\mathrm{k}}\beta _{\mathrm{k}}{{BM}}{\mathrm{pattern}}_{\mathrm{k,i,t}}$$

Where:

1. medication_i,t_ is a binary variable that is 1 for individual *i* on day *t* if a laxative is reported taken, else 0
2. symptom_j,i,t_ is an ordinal symptom severity indicator for individual *i* on day *t* with levels ranging from 0 to 4 for each of the *j* symptom types (e.g., gas, difficulty passing, etc.), with 0 indicating no symptoms and 4 indicating most severe symptoms
3. *BM*pattern_k,i,t_ is a dummy variable indicating if individual *i* reported being constipated, irregular, or “none of the above” as opposed to regular on day *t*, with regular BM pattern being the reference state

The HTML and PDF versions of the Article have been corrected.

